# Supplementary material for: Multi‐dataset identification of innovative feature genes and molecular mechanisms in keratoconus
Source: J Cell Mol Med. 2024 Sep 19;28(18):e70079. doi: 10.1111/jcmm.70079 (PMC11412914; doi:10.1111/jcmm.70079)
Supplement: Supplementary file 2 — Material S2. [file JCMM-28-e70079-s002.zip › 7.GSEA analysis/5.RNF217-AS1/KEGG_VIBRIO_CHOLERAE_INFECTION.html]

Details for gene set KEGG\_VIBRIO\_CHOLERAE\_INFECTION[GSEA]

|  || Dataset | RNF217-AS1.group.cls#h\_versus\_l |
| Phenotype | group.cls#h\_versus\_l |
| Upregulated in class | l |
| GeneSet | KEGG\_VIBRIO\_CHOLERAE\_INFECTION |
| Enrichment Score (ES) | -0.3138002 |
| Normalized Enrichment Score (NES) | -1.3059756 |
| Nominal p-value | 0.13709678 |
| FDR q-value | 0.39751276 |
| FWER p-Value | 0.947 |
Table: GSEA Results Summary

  

Fig 1: Enrichment plot: KEGG\_VIBRIO\_CHOLERAE\_INFECTION      
 Profile of the Running ES Score & Positions of GeneSet Members on the Rank Ordered List

  

| SYMBOL | TITLE | RANK IN GENE LIST | RANK METRIC SCORE | RUNNING ES | CORE ENRICHMENT || 1 | PRKCG | na | 203 | 0.657 | 0.1035 | No |
| 2 | SEC61A2 | na | 1167 | 0.422 | 0.1458 | No |
| 3 | ATP6V0D2 | na | 2490 | 0.317 | 0.1604 | No |
| 4 | TJP2 | na | 7866 | 0.131 | 0.0272 | No |
| 5 | ATP6V0D1 | na | 9204 | 0.101 | 0.0055 | No |
| 6 | KDELR1 | na | 10660 | 0.075 | -0.0239 | No |
| 7 | ACTB | na | 10680 | 0.075 | -0.0120 | No |
| 8 | KDELR2 | na | 11315 | 0.066 | -0.0193 | No |
| 9 | MUC2 | na | 11706 | 0.059 | -0.0207 | No |
| 10 | TJP1 | na | 12076 | 0.054 | -0.0223 | No |
| 11 | ATP6V1G3 | na | 12812 | 0.045 | -0.0361 | No |
| 12 | ACTG1 | na | 12835 | 0.044 | -0.0293 | No |
| 13 | PLCG1 | na | 13642 | 0.035 | -0.0468 | No |
| 14 | ATP6V0B | na | 15585 | 0.013 | -0.1006 | No |
| 15 | SEC61G | na | 15718 | 0.011 | -0.1025 | No |
| 16 | ATP6V1D | na | 15821 | 0.011 | -0.1037 | No |
| 17 | ATP6V0E1 | na | 16390 | 0.005 | -0.1193 | No |
| 18 | SEC61B | na | 16722 | 0.001 | -0.1286 | No |
| 19 | KDELR3 | na | 17204 | -0.004 | -0.1419 | No |
| 20 | ADCY9 | na | 17206 | -0.004 | -0.1413 | No |
| 21 | GNAS | na | 17807 | -0.009 | -0.1571 | No |
| 22 | ATP6V1F | na | 18311 | -0.014 | -0.1694 | No |
| 23 | ATP6V0C | na | 18707 | -0.017 | -0.1779 | No |
| 24 | ATP6V1G1 | na | 19140 | -0.021 | -0.1868 | No |
| 25 | ATP6V1C1 | na | 19502 | -0.025 | -0.1930 | No |
| 26 | ATP6V1B2 | na | 19557 | -0.026 | -0.1902 | No |
| 27 | ATP6V1E1 | na | 20165 | -0.032 | -0.2025 | No |
| 28 | ATP6V1A | na | 20315 | -0.033 | -0.2013 | No |
| 29 | ATP6AP1 | na | 20488 | -0.035 | -0.2005 | No |
| 30 | SLC12A2 | na | 21226 | -0.042 | -0.2147 | No |
| 31 | ATP6V1C2 | na | 21292 | -0.043 | -0.2094 | No |
| 32 | ARF1 | na | 21569 | -0.046 | -0.2097 | No |
| 33 | ATP6V1H | na | 21597 | -0.046 | -0.2028 | No |
| 34 | ATP6V0A1 | na | 21733 | -0.048 | -0.1987 | No |
| 35 | PDIA4 | na | 22484 | -0.056 | -0.2111 | No |
| 36 | ADCY3 | na | 22794 | -0.059 | -0.2101 | No |
| 37 | PRKACA | na | 23066 | -0.063 | -0.2075 | No |
| 38 | KCNQ1 | na | 23552 | -0.069 | -0.2100 | No |
| 39 | PRKCA | na | 23805 | -0.072 | -0.2052 | No |
| 40 | PLCG2 | na | 24806 | -0.087 | -0.2195 | No |
| 41 | SEC61A1 | na | 25161 | -0.093 | -0.2143 | No |
| 42 | PRKACB | na | 26457 | -0.116 | -0.2324 | No |
| 43 | CFTR | na | 28460 | -0.161 | -0.2634 | No |
| 44 | ATP6V1E2 | na | 30210 | -0.213 | -0.2783 | Yes |
| 45 | ATP6V0A4 | na | 30469 | -0.223 | -0.2487 | Yes |
| 46 | PRKX | na | 31362 | -0.257 | -0.2317 | Yes |
| 47 | ATP6V1G2 | na | 32923 | -0.355 | -0.2176 | Yes |
| 48 | ATP6V1B1 | na | 32937 | -0.356 | -0.1589 | Yes |
| 49 | ATP6V0E2 | na | 33133 | -0.370 | -0.1028 | Yes |
| 50 | PRKACG | na | 33174 | -0.374 | -0.0418 | Yes |
| 51 | PRKCB | na | 34175 | -0.520 | 0.0159 | Yes |
Table: GSEA details [plain text format]

  

Fig 2: KEGG\_VIBRIO\_CHOLERAE\_INFECTION      
 Blue-Pink O' Gram in the Space of the Analyzed GeneSet

  

Fig 3: KEGG\_VIBRIO\_CHOLERAE\_INFECTION: Random ES distribution      
 Gene set null distribution of ES for **KEGG\_VIBRIO\_CHOLERAE\_INFECTION**
